# WSES-AAST guidelines: management of inflammatory bowel disease in the emergency setting

**DOI:** 10.1186/s13017-021-00362-3

**Published:** 2021-05-11

**Authors:** Belinda De Simone, Justin Davies, Elie Chouillard, Salomone Di Saverio, Frank Hoentjen, Antonio Tarasconi, Massimo Sartelli, Walter L. Biffl, Luca Ansaloni, Federico Coccolini, Massimo Chiarugi, Nicola De’Angelis, Ernest E. Moore, Yoram Kluger, Fikri Abu-Zidan, Boris Sakakushev, Raul Coimbra, Valerio Celentano, Imtiaz Wani, Tadeja Pintar, Gabriele Sganga, Isidoro Di Carlo, Dario Tartaglia, Manos Pikoulis, Maurizio Cardi, Marc A. De Moya, Ari Leppaniemi, Andrew Kirkpatrick, Vanni Agnoletti, Gilberto Poggioli, Paolo Carcoforo, Gian Luca Baiocchi, Fausto Catena

**Affiliations:** 1 Department of Metabolic, Digestive and Emergency Minimally Invasive Surgery, Centre Hospitalier Intercommunal de Poissy et Saint Germain en Laye, 10 rue du Champ Gaillard, 78303 Poissy, France; 2 Addenbrooke’s Hospital, Cambridge University Hospitals NHS Foundation Trust, Cambridge, UK; 3 Department of General Surgery, University of Insubria, University Hospital of Varese, ASST Sette Laghi, Regione Lombardia, Varese, Italy; 4 RIMLS - Radboud Institute for Molecular Life Sciences, Radboud University-Nijmegen Medical Center, Nijmegen, The Netherlands; 5 Department of Trauma and Emergency Surgery, Parma University Hospital, Parma, Italy; 6 Department of General Surgery, Macerata Hospital, Macerata, Italy; 7 Scripps Memorial Hospital La Jolla, San Diego, California USA; 8 Department of Surgery, University Hospital of Pavia, Pavia, Italy; 9 Department of Emergency and Trauma Surgery, University Hospital of Pisa, Pisa, Italy; 10 Minimally Invasive and Robotic Digestive Surgery Unit, Regional General Hospital F. Miulli, Acquaviva delle Fonti (Bari), Italy; 11 Denver Health System - Denver Health Medical Center, Denver, USA; 12 Division of General Surgery, Rambam Health Care Campus, Haifa, Israel; 13 Department of Surgery, College of Medicine and Health Sciences, United Arab Emirates University, Al-Ain, United Arab Emirates; 14 First Clinic of General Surgery, University Hospital St George, Plovdiv, Bulgaria; 15 UCSD Health System - Hillcrest Campus Department of Surgery Chief Division of Trauma, Surgical Critical Care, Burns, and Acute Care Surgery, San Diego, CA USA; 16 Department of Colorectal Surgery, Portsmouth Hospitals NHS Trust, Hampshire, UK; 17 Government Gousia Hospital-Srinagar, Directorate of Health Services-Kashmir, Srinagar, Kashmir India; 18 Department of Abdominal Surgery, Umc Ljubljana, Ljubljana, Slovenia; 19 Department of Emergency Surgery, “A. Gemelli Hospital”, Catholic University of Rome, Rome, Italy; 20 Department of Surgical Sciences and Advanced Technologies, University of Catania, General Surgery, Cannizzaro Hospital, Catania, Italy; 21 Emergency Surgery Unit & Trauma Center, New Santa Chiara Hospital, University of Pisa, Pisa, Italy; 22 3rd Department of Surgery, Attikon General Hospital, National & Kapodistrian University of Athens (NKUA), Athens, Greece; 23 Department of Oncological Surgery “P.Valdoni”, Sapienza University, Rome, Italy; 24 Trauma/Acute Care Surgery Department, Medical College of Wisconsin/Froedtert Trauma Center, Wauwatosa, Wisconsin USA; 25 Department of Abdominal Surgery, Abdominal Center, University of Helsinki and Helsinki University Central Hospital, Helsinki, Finland; 26 General, Acute Care, Abdominal Wall Reconstruction, and Trauma Surgery Foothills Medical Centre, Calgary, Alberta Canada; 27 ICU Department, Bufalini Hospital, Cesena, Italy; 28 Department of Surgical Sciences, Policlinico Sant’Orsola Malpighi, Bologna, Italy; 29 Department of Surgery, University Hospital of Ferrara, Ferrara, Italy; 30 Department of Surgery, University of Brescia, Brescia, Italy

**Keywords:** Inflammatory bowel disease, Crohn’s disease, Ulcerative colitis, Emergency surgery, Perianal sepsis, Toxic megacolon, Peritonitis, Perforation, Percutaneous drainage, Abscess, SILS, Laparoscopy, Damage control surgery, Open abdomen

## Abstract

**Background:**

Despite the current therapeutic options for the treatment of inflammatory bowel disease, surgery is still frequently required in the emergency setting, although the number of cases performed seems to have decreased in recent years.

The World Society of Emergency Surgery decided to debate in a consensus conference of experts, the main pertinent issues around the management of inflammatory bowel disease in the emergent situation, with the need to provide focused guidelines for acute care and emergency surgeons.

**Method:**

A group of experienced surgeons and gastroenterologists were nominated to develop the topics assigned and answer the questions addressed by the Steering Committee of the project. Each expert followed a precise analysis and grading of the studies selected for review. Statements and recommendations were discussed and voted at the Consensus Conference of the 6th World Society of Emergency Surgery held in Nijmegen (The Netherlands) in June 2019.

**Conclusions:**

Complicated inflammatory bowel disease requires a multidisciplinary approach because of the complexity of this patient group and disease spectrum in the emergency setting, with the aim of obtaining safe surgery with good functional outcomes and a decreasing stoma rate where appropriate.

## Background

Inflammatory bowel disease (IBD) encompasses a group of chronic inflammatory disorders comprising most commonly of ulcerative colitis (UC) and Crohn’s disease (CD). The incidence of IBD appears to be rising in recent decades. Ng et al. [1] reported that the highest prevalence values were in Europe (UC 505 per 100 000 in Norway; CD 322 per 100 000 in Germany) and North America (UC 286 per 100 000 in the USA; CD 319 per 100 000 in Canada). The prevalence of IBD exceeded 0.3% in North America, Oceania, and many countries in Europe. Overall, the majority of studies on CD and UC report stable or decreasing incidence of IBD in North America and Europe. Since 1990, the incidence has been rising in newly industrialized countries in Africa, Asia, and South America, including Brazil [1]. The overall incidence of UC in Europe, North America, and Oceania is independent of gender. In CD, less consistent findings have been reported, with some cohorts suggesting a female predominance in the incidence of CD and others failing to find any gender difference. Differences in gender-specific incidence exist, with a female predominance in CD in western populations and a male predominance in eastern studies. No gender differences were found in UC [2].

IBD typically manifests in the 2nd or 3rd decade of life. Although their pathogenesis is still unclear, it is hypothesized that chronic intestinal inflammation originates from an overly aggressive mucosal immune response against luminal bacteria in genetically susceptible subjects.

CD is characterized by transmural inflammation that can occur in the entire gastrointestinal (GI) tract and common localizations include the terminal ileum and colon. Due to the transmural inflammation, complications may present such as abscesses and fistulas.

In contrast, UC demonstrates mucosal inflammation and typically starts distally in the rectum, showing progression towards the more proximal colon. The disease will mostly be limited to the colon and ileal involvement is rare (backwash ileitis).

Diagnosis of IBD is generally made by assessment of symptoms, biochemical markers, and colonoscopy combined with radiology and histology. The different phenotypes of IBD share common clinical features but may have a heterogeneous presentation which includes abdominal pain, vomiting, diarrhea, rectal bleeding, weight loss, and anemia. Extra-intestinal manifestations such as arthritis, skin disorders, and uveitis may also be present.

IBD is chronic and potentially disabling, frequently leading to hospitalizations, lower quality of life and inability to work, with a substantial socio-economic impact [3]**.**

IBD management aims to achieve induction of remission, followed by maintenance therapy to prevent recurrent disease flares.

IBD therapy is tailored and the choice of the treatment regimen depends on several factors including the type, distribution, and disease severity, as well as co-morbidity and patient preferences. Generally, depending on the level of severity, most patients with CD and to a lesser extent those with UC will require immunosuppression to control intestinal inflammation. Conventional immunosuppressive therapies include azathioprine, 6-mercaptopurine, methotrexate, and 6-thioguanine. These therapies may be necessary for many years, particularly given the incurable nature of CD.

In case of insufficient response to immunosuppressive treatment, or in case of intolerance, biologics are the next line of therapy in a step-up approach. Different mechanisms are currently available. Anti-TNF such as infliximab, adalimumab, and golimumab are available and usually the first biologic that is prescribed due to the lower costs since the introduction of biosimilars and good effectiveness/safety profile. Next line biologicals include vedolizumab (anti-integrin), preventing leukocyte homing to the gut, and ustekinumab for CD blocking the interleukin 12/23 pathway. Recently, tofacitinib was approved for the treatment of UC, which is a JAK inhibitor and belongs to the group of small molecules.

Despite the current therapeutic arsenal for the treatment of IBD, surgery is still frequently required although the number of cases performed seems to have decreased in recent years. It is reported that the risk of first CD surgery after 10 years of disease decreased from 44 to 21% in the last 2 decades in the UK [4], with the risk of a second resection decreasing from 40 to 17%. This is likely due to the introduction of anti-TNF therapy, as well as improved multidisciplinary IBD management aiding this development.

Similarly, colectomy rates in UC decreased in a prospective Swiss cohort and the 5-, 10-, 15-, and 20-year cumulative colectomy rates after diagnosis were 4.1%, 6.4%, 10.4%, and 14.4%, respectively [5]. Interestingly, the vast majority of colectomies took place within the first 10 years since diagnosis.

The improved outcomes for patients with CD are further reflected in recent studies. For example, the population-based cohort of South-Limburg (The Netherlands) showed that hospitalization rate reduced from 65.9% to 44.2% and the surgery rate from 42.9 to 17.4% at 5 years, respectively (both *P*<0.01) [6]. However, patients with CD still show progression towards a complicated phenotype. This is characterized by the formation of stenosis (stricturing phenotype) or abscess/fistula (penetrating phenotype). In contrast, patients who do not progress over time towards these phenotypes are considered “inflammatory phenotype.” The latter study showed that the rate of progression towards penetrating or stricturing phenotype was around 21% in the 1990s and this rate did not change until 2011 when 2 different time cohorts were analyzed. In contrast, the rate of immunosuppression increased from 30 to 70%, and biologic use from 3 to 41%.

Thus, despite improved IBD management and decreasing surgical rates, patients with complicated IBD continue to present with acute complications requiring admission for emergency care. This is in part explained by the progression towards a complicated phenotype (structuring or penetrating phenotype). Secondly, patients have more therapeutic options and continue to be treated with available biologics. When failure of biologic therapy occurs, patients are usually more refractory and prone to requiring hospitalization and surgery. Toxic colitis with or without megacolon, massive hemorrhage, free perforation, an acute abscess (either intra-abdominal or perianal) with sepsis, and intestinal obstruction are examples of acute surgical emergencies [7]***.***

CD can present with acute complications requiring emergency surgery in approximately 6–16% of cases [8]. In acute severe UC, intravenous corticosteroids remain the cornerstone of medical therapy but about 30% of patients do not respond to corticosteroids. After failing 3–5 days of corticosteroids, patients should be considered for second line medical therapy in the form of cyclosporine or anti-TNF therapy, as well as consideration and counselling for colectomy.

Complicated IBD requires a multidisciplinary approach because of the complexity of this group of patients. The management of IBD is very well established in the elective setting but is still unclear in the urgent/emergency setting with a lot of grey areas and a highly variable quality of management in the lack of established consensus and guidelines that could lead to poor overall and functional outcomes.

The World Society of Emergency Surgery (WSES) decided to debate in a consensus conference of experts in the fields, the main issues pertinent to the management of IBD in the emergent situation, with the need to provide a focused guide for acute care and emergency surgeons.

## Materials and methods

During the 2018 WSES congress, the Scientific Board of the WSES expressed the necessity to address the lack of guidelines about the management of IBD in the emergency setting, to improve outcomes, decreasing morbidity, and mortality correlated to the emergency treatment of these chronic and complex diseases.

A group of experienced surgeons and gastroenterologists were nominated to develop the topics assigned and answer the questions addressed by the Steering Committee (SC) of the project. The main topics debated are summarised in the Table 1. The scientific coordinator of the WSES IBD Guidelines supervised each step of literature searching, study selection, and the final presentation of evidence.

**Table 1:** Summary of topics and PICO questions

Topic	Question	Combination of words
**Initial assessment and Diagnosis**	Q.1: In patients with suspected complicated IBD, which are the appropriate biochemical investigations that should be performed?	“Crohn”, “Ulcerative colitis”, “abdominal pain”, “emergency”, “biochemical”, “laboratory”, “markers”, “investigation”; “test”; “metabolic panel”
**Initial assessment and Diagnosis**	Q.2: In patients with a suspected complicated IBD, which are the appropriate imaging studies that should be performed in the emergency setting?	“Crohn”; “Ulcerative colitis”; “emergency”; “radiology”; “computed tomography”; magnetic resonance”; ultrasonography”; “peritonitis”; abscess”; “occlusion”
**Non operative management and preoperative assessment**	Q.3: Which is the role of interventional radiology in the management of intra-abdominal abscesses related to Crohn’s disease in the emergency setting?	“Crohn"; “abscess”; “stricture”; “drainage”; “antibiotics”; “surgery”; “emergency”; “ulcerative colitis”
**Preoperative management**	Q.4: In patients presenting with complications related to IBD, what is the appropriate medical treatment and nutritional support? -The role of medical treatment and management of specific IBD drugs -The role of nutritional support	“Crohn”; "Ulcerative Colitis"; Nutritional support”; “immunosuppression”; “steroids”; “biologics”; “medical treatment” antibiotics”; “emergency”; “preoperative”; “postoperative”; “surgery”
** Non-operative vs Operative management** **Clinical setting:** **-Acute severe ulcerative colitis;** **-Toxic megacolon;** **-Uncontrolled bleeding;** **-Free perforation;** **-Intestinal obstruction**	Q.5: What are the indications for emergency surgery in patients presenting with complications related to IBD?	“Crohn”; "ulcerative colitis”; “toxic megacolon”; “upper gastrointestinal bleeding”;”peritonitis”; “perforation”; “occlusion”; “obstruction”;”emergency”; “surgery”; “indications”;”radiolology”;”angio-embolisation”; “computed tomography”;”angiography”, "lower gastrointestinal bleeding", "non operative management"
**Surgical management**	Q.6: Which surgical approach is recommended for complicated IBD in the emergency setting?	“Acute severe ulcerative colitis”; “intestinal bleeding”;”hemorrage”;“Crohn”; “anastomosis”; “laparoscopy”; “open”; “non operative management”; peritonitis”; “occlusion”; “perforation”; “toxic megacolon”; minimally invasive tecnique”; “emergency”;”damage control”;”open abdomen”
**Surgical management**	Q.7: How to manage perianal sepsis in the emergency setting?	“perianal”; “abscess”; “fistula”; “sepsis”;”antimicrobial”; “medical treatment”; “surgery”;”emergency”, "Crohn"

Each expert followed the PRISMA methodology [9] in the selection of papers to consider for review, and articles selected were included in the final analysis. Pediatric patients were excluded. The study group developed a focused draft and a variable number of statements. Each statement was evaluated according to the Grading of Recommendations, Assessment, Development and Evaluation (GRADE) [10].

The provisional statements and the supporting literature were reviewed and discussed with the WSES scientific coordinator by email and modified if necessary. The final data and contributions were presented at the 2019 WSES Congress at Nijmegen.

The WSES scientific coordinator of the project revised the statements, wrote the recommendations based on Consensus conference comments/suggestions, and wrote the final draft. It was submitted to all authors for evaluation and approval. All the comments and pertinent suggestions were considered in the final manuscript. Complicated IBD is defined as summarized in Table 2. Statements and recommendations are summarized in Table 3.

**Table 2:** Emergency complications in inflammatory bowel disease

Main acute complications Ulcerative Colitis	Main acute complications Crohn's Disease
Acute severe colitis	Acute severe colitis
Toxic megacolon	Toxic megacolon
Uncontrolled bleeding	Uncontrolled bleeding
Colonic perforation	Free perforation
	Abscess/fistula
	Intestinal obstruction

**Table 3:** Summary of statements and recommendations

***Initial assessment and diagnosis***	
**Q.1:** **In patients with suspected complicated IBD, which are the appropriate biochemical investigations that should be performed?**	
**Statement 1.1** In clinical practice, the diagnosis of Crohn’s Disease and Ulcerative Colitis is based on a set of modalities including clinical, biochemical, endoscopic, radiological, and histological diagnostics rather than a single reference standard (QoE low C). **Statement 1.2** In assessing an acute abdomen in patients with IBD, laboratory tests including full blood count, electrolytes, liver enzymes, inflammatory biomarkers such as erythrocyte sedimentation rate (ESR) and C Reactive Protein (CRP), serum albumin and pre-albumin (to assess nutritional status and degree of inflammation) are mandatory (QoE moderate B). **Statement 1.3** In case of a suspected IBD flare, infectious causes should be ruled out, especially *Clostridium difficile and Cytomegalovirus* (QoE low C).	
**Recommendation 1** We recommend assessing Crohn’s disease or Ulcerative colitis disease activity in the urgent clinical situation by performing the following laboratory tests: a full blood count, including haemoglobin, leukocytes count and platelet count; serum C-reactive protein level, erythrocyte sedimentation rate level, serum electrolytes, liver enzymes level, serum albumin, renal function and faecal calprotectin level, when it is possible. It’s mandatory to exclude any infectious diseases by performing blood-, stool cultures and toxin test for Clostridium difficile (Strong recommendation based on a moderate level of evidence 1B).	
**Q.2:** **In patients with a suspected complicated IBD, which are the appropriate imaging studies that should be performed in the emergency setting?**	
**Statement 2.1** Cross-sectional imaging (computed tomography, magnetic resonance imaging, ultrasonography) is recommended to detect strictures and extra-luminal IBD complications including fistulae and abscesses (QoE C). **Statement 2.2** Computed tomography and Magnetic resonance imaging are the most sensitive and specific imaging tests for detecting abscesses and stenosis in IBD (QoE B). **Statement 2.3** Contrast enhanced computed tomography is the key study in the emergency setting in assessing IBD extra-luminal complications such as abscesse and fistulae, and a source of bleeding in the case of gastro-intestinal haemorrhage (QoE B). **Statement 2.4** The diagnostic accuracy of magnetic resonance enterography for assessing disease activity and complications related to IBD (including strictures) is similar to CT scan with a decreased ionising radiation exposure (QoE C) **Statement 2.5** Point of Care ultrasonography can have a role in showing free fluid, abscesses or intestinal distention in the emergency department, particularly when CT scan is not available (QoE C) **Statement 2.6** Sigmoidoscopy allows intra-luminal assessment of distal IBD disease activity, bleeding source identification and biopsies in an acute setting, when it is available (QoE C). **Statement 2.7** In stable patients presenting with signs of gastrointestinal haemorrhage, computed tomography angiography should be considered to localise the bleeding site before angio-embolisation or surgery, especially when endoscopic assessment is not available (QoE C)	
**Recommendations 2** We recommend investigating the acute abdomen in IBD patients with IV contrast-enhanced computed tomography scan in the emergency setting, to exclude the presence of intestinal perforation, stenosis, bleeding and abscesses and to help guide decision making for immediate surgery or initial conservative management (Strong recommendation based on low level evidence 1C). We suggest performing a point of care ultrasonography (if skills are available) when computed tomography scan is not available, in order to assess the presence of free intra-abdominal fluid, intestinal distension or abscess. The magnetic resonance enterography, (if available) is the preferred technique to diagnose strictures, to differentiate fibrotic from inflammatory components and disease activity (Weak recommendation based on low level evidence 2C). In stable patients presenting with signs of gastrointestinal bleeding, we recommend performing a computed tomography angiography to localise the bleeding site before angio-embolisation or surgery (Weak recommendation based on low level evidence 2C). If computed tomography and ultrasonography are unavailable, we suggest referring stable patients to a hospital where 24/7 emergency imaging is available (Weak recommendation based on very low level evidence 2D)	
***Preoperative management and non operative management***	
**Q.3:** **Which is the role of interventional radiology in the management of intra-abdominal abscesses related to Crohn’s disease in the emergency setting?**	
**Statement 3.1** Percutaneous drainage associated with antimicrobial treatment should be considered as first line treatment in the management of abscesses related to Crohn’s disease, in stable patients (QoE C). **Statement 3.2** Small abscesses (< 3 cm) could be treated with intravenous antibiotics with a risk of recurrence, especially if associated with enteric fistula (QoE B) **Statement 3.3** Percutaneous drainage of abscesses > 3 cm could avoid immediate surgery and should be used as a bridging procedure before elective surgery to reduce the need for stoma creation and limit intestinal resection in malnourished and high risk patients (QoE C). **Statement 3.4** Surgery should be considered in the case of failure of percutaneous drainage and in patients with signs of septic shock (QoE C). **Statement 3.5** Surgery should be considered for patients with enteric fistulae and if clinical evidence of sepsis persists despite the initial treatment plan (QoE C).	
**Recommendations 3** We recommend performing radiological percutaneous drainage of intra-abdominal abscesses > 3 cm related to Crohn’s disease associated with early empiric administration of antibiotics, to adapt these as soon as possible to microbiological cultures results. The antimicrobial therapy should be re-evaluated according to patient’s clinical and biochemical features (Strong recommendation based on a low level evidence 1C). We recommend administering an early empiric antimicrobial therapy in stable patients presenting with abscess < 3 cm, with close clinical and biochemical monitoring (Strong recommendation based on a low level evidence 1C).	
**Q.4:** **In patients presenting with complications related to IBD, what is the appropriate medical treatment and nutritional support?**	
***a) The role of medical treatment and management of specific IBD drugs***	
**Statement 4.1** The optimal management of IBD patients presenting with acute abdominal pain is multidisciplinary, involving a gastroenterologist and an acute care surgeon (QoE C). **Statement 4.2** All IBD patients presenting with an acute abdomen should receive adequate volume of intravenous fluids, low-molecular-weight heparin for thromboprophylaxis and electrolyte abnormalities and anaemia should be corrected (QoE C). **Statement 4.3** Antibiotics should not be routinely administered, but only if superinfection is considered and in the presence of an intra-abdominal abscess (QoE B). **Statement 4.4** In case of superinfection or abscesses, prompt antimicrobial therapy against Gr/−/ aerobic and facultative bacilli and Gr/+/*streptococci* and obligate anaerobic bacilli is needed according to the epidemiology and resistance of the setting. Antimicrobial therapy duration depends on the patient’s clinical feature and laboratory tests results such as serum CRP level. (QoE **A)** **Statement 4.5** The initial medical treatment for severe active UC is intravenous corticosteroids, in case of hemodynamic stability of the patient (QoE A). **Statement 4.6** The response to intravenous steroids should be best assessed by the third day (QoE C). **Statement 4.7** In non-responder hemodynamically stable patients, medical rescue therapy including infliximab in combination with a thiopurine, or ciclosporin should be considered in a multidisciplinary approach (QoE B). **Statement 4.8** Infliximab should be considered if anti-inflammatory therapy for penetrating ileocecal Crohn’s disease is required, following adequate resolution of intra-abdominal abscesses in a multidisciplinary approach (QoE C). **Statement 4.9** Preoperative treatments with immunomodulators associated with anti-TNF-α agents and steroids are risk factors for intra-abdominal sepsis in patients requiring emergency resectional surgery (QoE B) **Statement 4.10** In complex perianal fistulizing disease infliximab or adalimumab can be used as first line therapy in combination with azathioprine following adequate surgical drainage if indicated. A combination of ciprofloxacin and anti-TNF improves short term outcomes (QoE A).	
***b) The role of nutritional support***	
**Statement 4.11** Preoperative nutritional support is mandatory in severely undernourished patients (QoE A) **Statement 4.12** Total Parenteral nutrition should be reserved for nutritionally deficient IBD patients unable to tolerate enteral nutrition and when the enteral route is contraindicated, in critically ill patients presenting with signs of shock, intestinal ischemia, high output fistula, and/or severe intestinal haemorrhage (QoE B) **Statement 4.13** Total parenteral nutrition is the mode of choice when emergency surgery is needed for complicated IBD (QoE A)	
**Recommendations 4** We recommend evaluating medical treatment in IBD patients presenting with acute abdominal pain and disease activity in a multidisciplinary approach (Strong recommendation based on low level evidence 1C). We recommend not routinely administrating antibiotics in IBD patients but only in the presence of superinfection, intra-abdominal abscesses, and sepsis (Strong recommendation based on high level evidence1A) We recommend administering antibiotics according to the epidemiology and resistance of the setting in a duration that depends on the patient’s clinical and biolchemical findings. Antifungals should be reserved for high risk patients such as those with bowel perforation and recent steroid treatment. (Strong recommendation based on high level evidence 1A) We recommend administering as soon as possible venous thromboembolism prophylaxis with LMWH for the high risk of thrombotic events related to complicated IBD and the emergency setting (Strong recommendation based on high level evidence 1A) We recommend weaning off steroids (wean preoperatively, ideally 4 weeks) and stopping immunomodulators associated with anti-TNF-α agents before surgery, as soon as possible to decrease the risk of postoperative complications, in accordance with a gastroenterologist (Strong recommendation based on moderate level evidence 1B) We recommend administering nutritional support (parenteral or enteral, according to GI function and in conjunction with a dietician/nutrition team) in IBD patients as soon as possible (Strong recommendation based on moderate level evidence 1B)	
***Non Operative*** **vs** ***Operative management***	
**Q.5: What are the indications for emergency surgery in patients presenting with complications related to IBD?**	
Urgent surgical treatment is to be considered in the following clinical setting:	
**1) ACUTE SEVERE ULCERATIVE COLITIS**	
**Statement 5.1.1** If a patient’s condition does not improve or deteriorates within 48 to 72 h from initiation of medical therapy, in acute severe ulcerative colitis second-line therapy or surgery should be considered and discussed by the emergency surgeon and the gastroenterologist (QoE C) **Statement 5.1.2** In the event of surgical complications such as free perforation, life-threatening haemorrhage (unstable patients) or generalised peritonitis, immediate surgery is recommended in acute severe ulcerative colitis (QoE B) **Statement 5.1.3** In case of no improvement with second line therapy, in discussion with the gastroenterologist, surgery is recommended in acute severe ulcerative colitis (QoE C) **Statement 5.1.4** Subtotal colectomy with ileostomy is a safe and effective treatment for patients requiring emergency surgery for acute severe ulcerative colitis presenting with massive colorectal haemorrhage (QoE B)	
**Recommendations 5.1** We suggest evaluating all hemodynamically stable patients presenting with acute severe ulcerative colitis in a multidisciplinary approach with the gastroenterologist to decide on options for initial medical treatment (Weak recommendation based on low level evidence 2C) We recommend performing emergency surgical exploration in hemodynamically unstable patients, according to damage control principles and in patients presenting with colonic perforation. Subtotal colectomy with ileostomy is the surgical treatment of choice in patients acute severe ulcerative colitis patients presenting massive colorectal haemorrhage or non responders to medical treatment (Strong recommendation based on high level evidence 1A)	
**2) TOXIC MEGACOLON**	
**Statement 5.2.1** In patients presenting with toxic megacolon complicated by perforation, massive bleeding (unstable patients), clinical deterioration and signs of shock, surgery is mandatory (QoE A). **Statement 5.2.2** In patients presenting with toxic megacolon, showing no clinical improvement and biological signs of deterioration after 24–48 h of medical treatment, surgery is mandatory (QoE B). **Recommendation 5.2** We recommend not delaying surgery in critically ill patients presenting with toxic megacolon (Strong recommendation based on low level evidence 1C)	
**3) UNCONTROLLED GASTROINTESTINAL BLEEDING**	
**Statement 5.3.1** Pre-operative localisation of the bleeding site, with the aim of excluding an upper gastrointestinal or an anorectal bleeding may allow better planning the surgical strategy (QoE C). **Statement 5.3.2** An upper and **l**ower GI endoscopy should be the initial diagnostic procedure for nearly all stable patients presenting with acute gastro-intestinal bleeding (QoE C). **Statement 5.3.3** Computed tomography angiography should be performed in patients with ongoing bleeding who are hemodynamically stable after resuscitation (QoE C). **Statement 5.3.4** Surgical treatment is recommended in patients with life-threatening bleeding and persistent hemodynamic instability and in patients with acute severe ulcerative colitis non-responders to medical treatment presenting with a massive colorectal haemorrhage (QoE B). **Statement 5.3.5** Significant recurrent gastrointestinal bleeding could be an indication for urgent surgery (QoE C).	
**Recommendations 5.3** We recommend performing immediate surgery in unstable patients presenting with hemorrhagic shock, and non responders to resuscitation. An intra-operative ileoscopy, if available, could be useful in localising the bleeding source in patients with Crohn’s disease. In patients presenting with acute severe ulcerative colitis and refractory haemorrhage, non responders to medical treatment, the surgical treatment of choice is a subtotal colectomy with ileostomy, if skills are present (Strong recommendation based on low level evidence 1C). We suggest evaluating hemodynamically stable IBD patients presenting with a gastrointestinal bleeding at first with a sigmoidoscopy and an esophagogastroduodenoscopy (Weak recommendation based on low level evidence 2C)	
**4) FREE PERFORATION**	
**Recommendation 5.4** We recommend performing surgical exploration in the presence of radiological signs of pneumoperitoneum and free fluid within the peritoneal cavity in acutely unwell patients presenting with complicated Crohn’s disease or acute severe ulcerative colitis (Strong recommendation based on low level evidence 1C)	
**5) INTESTINAL OBSTRUCTION**	
**Statement 5.5.1** Surgery is mandatory for symptomatic intestinal strictures that do not respond to medical therapy and are not amenable to endoscopic dilatation in Crohn’s disease (QoE C). **Statement 5.5.2** Any colorectal stricture should be assessed with endoscopic biopsies to ensure the absence of malignancy (QoE C).	
**Recommendation 5.5** We recommend performing surgery in patients presenting with small bowel obstruction because of fibrotic or medically-resistant stenosis (Strong recommendation based on low level evidence 1C)	
**Surgical management**	
**Q.6:** **Which surgical approach is recommended for complicated IBD in the emergency setting?**	
**1) Emergency Surgery for ulcerative colitis**	
**Statement 6.1.1** In the setting of free perforation and generalised peritonitis or toxic megacolon, in hemodynamically unstable patient, an open approach is recommended (QoE C). **Statement 6.1.2** Both open and laparoscopic approaches are otherwise appropriate in the emergency setting, according to patient’s haemodynamic stability and signs of sepsis in complicated ulcerative colitis (QoE C). **Statement 6.1.3** A laparoscopic approach (multi-port or in a single incision), if local expertise allows, may reduce length of stay and morbidity in hemodynamically stable patients with complicated ulcerative colitis (QoE C)	
**2) Emergency Surgery for Crohn’s Disease**	
**Clinical scenarios:**	
**a) Intestinal obstruction**	
**Statement 6.2.1** If emergency surgery is indicated, a laparoscopic approach to adhesiolysis and bowel resection is recommended if appropriate expertise exists, with care taken to avoid iatrogenic bowel injury in patients presenting intestinal obstruction in Crohn’s disease (QoE C)	
**b) Bleeding**	
**Statement 6.2.2** If the patient presenting with gastrointestinal bleeding in Crohn’s disease is haemodynamically stable and endoscopic and/or interventional radiology measures have been unsuccessful, then a surgical exploration in a laparoscopic (multi-port or in single incision) approach is recommended. (QoE C) **Statement 6.2.3** If the patient presenting with gastrointestinal bleeding in Crohn’s disease is haemodynamically unstable and endoscopic and/or interventional radiology procedures have been unsuccessful, then a surgical exploration in an open approach is recommended to reduce operating time (QoE C**).**	
**c) Free perforation and purulent/faecal peritonitis**	
**Statement 6.2.4** A laparoscopic approach with resection, lavage and stoma is suggested in hemodynamically stable patients presenting with perforation and peritonitis in Crohn’s disease, to avoid complications associated with anastomotic leak (QoE C) **Statement 6.2.5** If there is haemodynamic stability and only localised contamination, an anastomosis may be considered but other factors will also need to be considered (QoE C) **Statement 6.2.6** If evidence of severe sepsis/septic shock, damage control surgery may be considered, with resection, stapled off bowel ends and temporary closure (laparostomy) with return to theatre in 24–48 h for a second look, washout and consideration of stoma vs anastomosis (QoE C).	
**d) Crohn’s Colitis**	
**Statement 6.2.7** Subtotal colectomy and ileostomy is the emergency operation of choice for severe acute and refractory colitis, with open and laparoscopic approaches appropriate in the emergency setting, according to hemodynamic patient’s stability (QoE C). **Statement 6.2.8** A laparoscopic approach, if local expertise allows, may reduce length of hospital stay and risk of infectious complications (QoE C). **Statement 6.2.9** There is insufficient evidence to recommend SILS or robotic surgery in the emergency setting (QoE C).	
**3) Anastomotic considerations in emergency surgery for Crohn’s Disease**	
**Statement 6.3.1** If a patient in the emergency setting has 2 or more risk factors for anastomotic complications, then a stoma should be formed following resection. (QoE C) **Statement 6.3.2** If a decision to anastomose has been made, there is no evidence to suggest that one type of anastomosis (stapled vs hand sewn) is superior to the other in terms of complication rates or recurrence, and the decision can be left to surgeon preference (QoE C).	
**Recommendations 6** We recommend performing a surgical exploration by laparotomy in a hemodynamically unstable patient presenting with complications related to IBD such as perforation and severe peritonitis, massive intestinal bleeding, obstruction, toxic megacolon, severe colitis non responder to medical treatment, taking in to consideration damage control surgery principles with or without an open abdomen (Strong recommendation based on low level evidence 1C). We recommend performing a laparoscopic approach in hemodynamically stable patients presenting with complications related to IBD, when skills are available, in order to decrease morbidity and length of hospital stay (Strong recommendation based on low level evidence 1C). We recommend performing a subtotal colectomy with ileostomy in patients presenting with acute severe refractory colitis, and massive colorectal bleeding non responders to medical treatment, in a laparoscopic or open approach according to patient’s hemodynamic stability and surgeon’s skill (Strong recommendation based on low level evidence 1C). We suggest considering an (stapled or hand sewn) anastomosis in hemodynamically stable patients with Crohn’s disease who have good pre-existing nutritional status and who are taking no steroids or other immunosuppression and presenting with no bowel vascular compromise and only localised peritonitis. A defunctioning stoma should also be considered in the emergency setting. (Weak recommendation based on low level evidence 2C)	
**Q.7:** **How to manage perianal sepsis in the emergency setting?**	
**Statement 7.1** An acute abscess should be adequately drained under general anaesthetic, with no routine requirement for wound packing. (QoE C) **Statement 7.2** No active attempt should be made to find an associated anal fistula at the initial abscess presentation. (QoE C) **Statement 7.3** If an obvious fistula exists (without probing), the fistula should not be laid open and a loose draining seton should be inserted (QoE C) **Statement 7.4** There is no role for any additional surgical fistula treatment modality in the emergency treatment of Crohn’s perianal sepsis. (QoE C) **Statement 7.5** An assessment of the rectum should be made at the time of abscess drainage, to assess for signs of proctitis. (QoE C)	
**Recommendation 7** We recommend performing adequate surgical drainage of perianal abscess in Crohn’s disease without searching for an associated fistula (Strong recommendation based on low level evidence 1C)	

Clinicians and surgeons should be aware that these guidelines should be considered as an adjunctive tool for decision and management but they do not substitute for the clinical judgment for individual patients.

## Results

### Q.1: In patients with suspected complicated IBD, which are the appropriate biochemical investigations that should be performed?

#### Statement 1.1

In clinical practice, the diagnosis of CD and UC is based on a set of modalities including clinical, biochemical, endoscopic, radiological, and histological diagnostics rather than a single reference standard (QoE low C).

#### Statement 1.2

In assessing an acute abdomen in patients with IBD, laboratory tests including full blood count, electrolytes, liver enzymes, inflammatory biomarkers such as erythrocyte sedimentation rate (ESR) and C-reactive protein (CRP), and serum albumin and pre-albumin (to assess nutritional status and degree of inflammation) are mandatory (QoE moderate B).

#### Statement 1.3

In case of a suspected IBD flare, infectious causes should be ruled out, especially *Clostridium difficile and Cytomegalovirus* (QoE low C).

#### Recommendation

We recommend assessing Crohn’s disease or ulcerative colitis disease activity in the urgent clinical situation by performing the following laboratory tests: a full blood count, including hemoglobin, leukocyte count, and platelet count; serum C-reactive protein level, erythrocyte sedimentation rate; serum electrolytes; liver enzymes level; serum albumin; renal function; and fecal calprotectin level, when it is possible. It is mandatory to exclude any infectious diseases by performing blood-, stool cultures, and toxin test for Clostridium difficile (strong recommendation based on a moderate level of evidence 1B).

#### Summary of evidence and discussion

In assessing a patient with acute abdominal pain in the emergency room, the main laboratory tests requested are full blood count (20.1%), electrolytes (19.1%), cardiac enzymes (19.0%), and liver function tests (11.5%) [11].

In differentiating the cause underlying acute abdominal pain, the diagnostic accuracy values of C-reactive protein (CRP) and white blood cell count (WBC) can be elevated [12].

IBD disease activity will usually impact laboratory tests results with anemia, leukocytosis, thrombocytosis, elevated liver enzymes, hypoalbuminemia, and increased inflammatory markers. In addition, therapies may cause abnormalities in liver enzymes, leukocytes, and kidney function. Consequently, for patients with IBD admitted to the emergency room for evaluation, laboratory tests should always include full blood count with differential, comprehensive metabolic panel, liver enzymes, and lipase.

CRP and fecal calprotectin (FC) are the most widely used biomarkers for IBD evaluation. CRP is the inflammatory marker of choice as it is more sensitive than erythrocyte sedimentation rate (ESR) for the evaluation of acute abdominal pain in patients with IBD, and correlates better with endoscopic disease activity in CD rather than in UC [13, 14]. It should be noted that a normal CRP does not rule out CD disease activity; therefore, the results should be interpreted with caution given the low sensitivity of this test.

The sensitivity of CRP ranges from 70 to 100% in the differential diagnosis between CD versus irritable bowel syndrome and ranges from 50 to 60% in UC [15]. Levels of CRP are higher in active CD than in UC [16].

ESR determination monitors satisfactorily the acute-phase response of IBD after the first 24 h. In contrast, during the first 24 h, the CRP is a better indicator of the acute phase. The ESR, compared with CRP, reaches the highest point less quickly, and it decreases more slowly and has a lesser degree of change [17].

Previous studies assessing the best monitoring of medical treatment measured prospectively some laboratory parameters such as full blood count, CRP, ESR, alfa1 antitrypsin, and orosomucoid in CD patients every 6 weeks after recent weaning of steroids [17] and showed that the best predictor of short-term relapse is the combination of CRP and ESR. Patients with CRP > 20 mg/L and ESR > 15 mm had an eight-fold increased risk of relapse with a negative predictive value of 97%, suggesting that normal CRP and ESR could almost exclude relapse in the next 6 weeks.

Anti-inflammatory or immunosuppressive drugs do not affect CRP production. Therefore, changes of CRP concentrations during treatment occur only as a result of the effect of the drug on the inflammation or disorder.

In addition, in assessing acute severe UC, defining the population by Truelove Witts criteria (summarized in Table 4) is essential [18]. The Truelove-Witts criteria combine frequency of bloody stools (⩾6 per day) with at least one marker of systemic toxicity such as pulse rate >90 bpm, temperature >37.8°C, hemoglobin <10.5 g/dl, and/or an ESR >30 mm/h. In patients with UC, the risk of progression to the second-line therapy is directly dependent on the number of variables present on admission, with a 50% risk for colectomy when three or more additional criteria are present [19]. After 3 days of intensive treatment (hydrocortisone and/or cyclosporine/anti-TNF) patients with frequent stools (> 8/day), or 3–8 stools/day and CRP > 45 mg/L, should be identified and reviewed jointly by a gastroenterologist and surgeon as most of them will need to undergo colectomy [19].

**Table 4:** Classification of ulcerative colitis per Truelove and Witts criteria at admission

Variables	Mild	Moderate	Severe
Stool frequency/day	≤4 per day	4–6 per day	≥6 per day
Blood in stool	None or small	-	Present
Temperature	Apyrexial	Intermediate	>37.8
Heart rate bpm	<90min/min	Intermediate	>90/min
Anemia (Hb=g/dL)	>11	10.5–11	<10.5
Erythrocite sedimentation rate (mm/hour)	<20	20–30	>30mm/h

Thrombocytosis correlates well with IBD disease severity, and, interestingly, it may persist even after bowel resection in some patients with IBD. The mean platelet volume has been proposed as a potential marker of clinical disease activity, being inversely proportional to the levels of CRP and ESR. The cause of the reduction in platelet volume in clinically active UC is unknown, but it may be a direct result of the thrombopoiesis disorder often observed in the early phases of systemic inflammatory progression [20]. The platelets also relate to the increased incidence of thromboembolic phenomena in CD and UC. Some studies report that spontaneous platelet aggregation is observed in more than 30% of patients with IBD [21].

The number of white blood cells (WBC) increases during the acute phase response, and it is influenced by immunosuppressive drugs utilized in IBD, such as glucocorticoids (increased WBC) or azathioprine and 6-mercaptopurine (decreased WBC).

Serum albumin is a negative acute phase marker and decreased levels may be found during inflammation [16].

Fecal calprotectin (FC) is a granulocyte-derived protein measured in the stool and is a non-invasive, cheap, and extensively studied biomarker used in IBD which correlates with clinical and endoscopic disease activity [14]. A cutoff of 30 μg/g had 100% sensitivity in discriminating active CD from irritable bowel syndrome in the study of Tibble and colleagues [22]

The correlation with disease activity is less robust for disease localised to the terminal ileum (versus distal colonic disease), with likelihood of false negative results in case of proximal disease. Most hospitals will not have an immediate assay ready for same-day results, so this can limit the application of this marker in an emergency setting.

In a patient with diarrhoea, stool cultures should be obtained. In particular, fever and sudden onset of symptoms may direct the differential diagnosis towards an infection. In the latter setting, bacterial stool culture or PCR, and especially *C. difficile* toxin, must be considered. IBD patients are at increased risk for *C difficile* and subsequent hospitalization and colectomy [23]. In addition, corticosteroids seem to be an independent risk factor for presenting with infectious colitis [24]. Depending on the clinical setting, PCR for viral and parasitic agents may be considered. For example, IBD patients receiving immunosuppression are more prone to CMV colitis, which can be measured in serum and biopsies [25]. Blood cultures are mandatory.

Clinical evaluation and laboratory tests are useful to stratify IBD patients in to low and high risk of complicated disease.

Khoury et al. [26] developed a diagnostic clinical score to predict the presence of an intra-abdominal abscess in CD patients presenting with acute abdominal pain in the ED. This score included 5 parameters that were significantly associated with abscess formation, such as ileo-colonic location of the disease, perianal CD, neutrophil-to-lymphocyte ratio, and CRP level, whereas the current use of corticosteroids was negatively associated with abscess formation.

### Q.2: In patients with a suspected complicated IBD, which are the appropriate imaging studies that should be performed in the emergency setting?

#### Statement 2.1

Cross-sectional imaging (computed tomography, magnetic resonance imaging, ultrasonography) is recommended to detect strictures and extra-luminal IBD complications including fistulae and abscesses (QoE C).

#### Statement 2.2

Computed tomography and magnetic resonance imaging are the most sensitive and specific imaging tests for detecting abscesses and stenosis in IBD (QoE B).

#### Statement 2.3

Contrast-enhanced computed tomography is the key study in the emergency setting in assessing IBD extra-luminal complications such as abscesse and fistulae, and a source of bleeding in the case of gastro-intestinal haemorrhage (QoE B).

#### Statement 2.4

The diagnostic accuracy of magnetic resonance enterography for assessing disease activity and complications related to IBD (including strictures) is similar to CT scan with a decreased ionising radiation exposure (QoE C)

#### Statement 2.5

Point of care ultrasonography can have a role in showing free fluid, abscesses, or intestinal distention in the emergency department, particularly when CT scan is not available (QoE C)

#### Statement 2.6

Sigmoidoscopy allows intra-luminal assessment of distal IBD disease activity, bleeding source identification, and biopsies in an acute setting, when it is available (QoE C).

#### Statement 2.7

In stable patients presenting with signs of gastrointestinal hemorrhage, computed tomography angiography should be considered to localize the bleeding site before angio-embolization or surgery, especially when endoscopic assessment is not available (QoE C)

#### Recommendations

We recommend investigating the acute abdomen in IBD patients with IV contrast-enhanced computed tomography scan in the emergency setting, to exclude the presence of intestinal perforation, stenosis, bleeding, and abscesses and to help guide decision making for immediate surgery or initial conservative management (strong recommendation based on low-level evidence 1C).

We suggest performing a point of care ultrasonography (if skills are available) when computed tomography scan is not available, in order to assess the presence of free intra-abdominal fluid, intestinal distension, or abscess. The magnetic resonance enterography (if available) is the preferred technique to diagnose strictures, to differentiate fibrotic from inflammatory components and disease activity (weak recommendation based on low-level evidence 2C).

In stable patients presenting with signs of gastrointestinal bleeding, we recommend performing a computed tomography angiography to localize the bleeding site before angio-embolization or surgery (weak recommendation based on low-level evidence 2C).

If computed tomography and ultrasonography are unavailable, we suggest referring stable patients to a hospital where 24/7 emergency imaging is available (weak recommendation based on very low-level evidence 2D)

#### Summary of evidence and discussion

An accurate acute abdominal assessment in patients in the emergency room with IBD is crucial to aid an early diagnosis and optimal treatment plan. Symptoms may result from the underlying IBD, or disease complications, but can also reflect a complication of therapy, an infection, or a separate medical problem. Imaging studies are mandatory to assess disease phenotype and complications, in order to facilitate informed decision making.

Usually, a cross-sectional study involving ultrasound (US), computed tomography (CT) or magnetic resonance imaging (MRI), allows for a full thickness evaluation of the bowel wall and associated abnormalities. The potential benefits of cross-sectional imaging in patients with IB include better inflammation grading, such as identification of mild degree of activity, which may be relevant whenever assessing response to treatment and, of utmost importance, an accurate preoperative detection and grading of fibrosis in stricturing CD, facilitating surgical versus medical therapeutic decisions.

The Truelove-Witts classification for UC and the Montreal classification (summarized in Tables 4 and 5) to assess CD phenotype are frequently used to stratify IBD patients at admission, and they require laboratory test and abdominal imaging results.

**Table 5:** Montreal classification for Crohn’s disease phenotype

Age at diagnosis (A)	Location of disease (L)	Behavior of disease (B)
A1: ≤16 years A2: 17–40 years A3: >40 years	L1: ileal L2: colonic L3: ileocolonic L4: modifier for upper gastrointestinal tract	B1: non-stricturing/nonpenetrating B2: stricturing B3: penetrating p: modifier for perianal disease

It is fundamental in an emergency to check the IBD disease activity and extent. In CD, the disease behavior can progress towards a penetrating phenotype over time, and in UC, can involve all of the colon.

In the emergency setting*,* CT should be the first radiological investigation to assess the acute abdomen in this group of patients, especially in the case of a suspected intra-abdominal abscess, perforation, or intestinal obstruction due to stricture(s). Using surgery as a reference standard, CT showed a sensitivity of 85% and a specificity of 88% for the detection of intra-abdominal abscesses [27].

Moreover, it is useful to stratify patients for immediate surgery or a medical treatment plan in assessing the type of bowel strictures; in fact, inflammatory strictures could benefit from a medical anti-inflammatory treatment, and fibrotic strictures could require endoscopic balloon dilation or surgery.

US showed specificity and sensitivity of 86% and 94% in detecting small bowel inflammation in comparison with MRI sensitivity and specificity that is 74% and 91%, respectively, in expert hands [28, 29].

A systematic review [30] showed that conventional trans-abdominal US sensitivity for stricture diagnosis ranged from 80 to 100% with specificity rates of 63–75%. The application of small intestinal contrast US demonstrated increased sensitivity rates of 88–98% with specificity rates ranging from 88 to 100%. CT enterography (CTE) sensitivity and specificity were reported to be both 100%. CT enteroclysis, in which the luminal contrast is delivered direct to the small bowel, had a sensitivity of 92% and specificity of 39% reported in one study only. With regard to magnetic resonance imaging enterography (MRE), the sensitivity for stricture detection ranged from 75 to 100% with specificity between 91% and 96% [30].

In a prospective blinded study, Point of Care US (POCUS) demonstrated that it is an accurate technique in defining disease activity and extent in IBD compared to ileocolonoscopy with the advantage of being non-invasive. It showed a 91% sensitivity and 83% specificity for detecting endoscopically active IBD, correlating with a positive predictive value (PPV) of 89%, a negative predictive value (NPV) of 86%, and a kappa coefficient of 0.74 (88%). POCUS-defined disease extent has a 87% sensitivity and 81% specificity, correlating with a PPV of 85% and NPV of 83% and a kappa coefficient of 0.70 (85%) [31].

Strictures can be assessed reliably by both CT and MRI. Sensitivity was 85% vs 92% and specificity was 100% vs 90%, respectively [32]. In addition, CTE is a modality that can be applied and it will provide a more detailed assessment of the bowel wall. However, the need for large volume oral contrast prohibits its use in the emergency setting.

In a prospective cohort study (31 participants), Mao et al. demonstrated that intra-cavitary contrast-enhanced US (IC-CEUS) could be a valid, radiation-free, safe, optional method to detect a fistulous tract associated with abscesses in CD patients with a sensitivity and specificity of 86.7% (95% confidence interval [CI], 68.4–95.6%) and 100% [95% CI, 5.5–100.0%], respectively. Moreover, authors reported that combining IC-CEUS and CTE/magnetic resonance imaging enterography (MRE), the fistula/sinus tract was clearly demonstrated in 29 patients [93.5%, 29/31]. The mean duration of the IC-CEUS procedure was 8.6 min [range 5.0–12.0] [33]. However, this technique is not widely used.

In clinical practice, MRE is frequently used in the outpatient setting. The lack of radiation and the excellent quality of images are advantages of this technique. This is particularly applied for evaluation of the small bowel and perineum. However, the use in an emergency setting is limited due to the oral contrast, increased study time, costs, and lack of availability.

In stable patients presenting with signs of GI hemorrhage, CT angiography should be considered to localize the bleeding site before angio-embolization or surgery, when an endoscopic evaluation is not possible and the patient is unable to tolerate the bowel preparation. A systematic review showed high sensitivity (85.2%) and high specificity (92.1%) of CT angiography for diagnosing acute gastrointestinal bleeding [34].

Endoscopy may be of added value as a diagnostic procedure for selected patients with IBD presenting with lower GI-bleeding in an emergency setting. A full colonoscopy is usually not possible given the need for oral bowel preparation prior to the colonoscopy, as well as the inability for oral intake of large volumes. However, a flexible sigmoidoscopy is possible with preparation with an enema. This procedure can aid in establishing the level and location of disease activity in UC and distal colonic CD and to detect preoperatively a source of bleeding. In addition, it can be used to rule out other conditions such as colonic ischemia, infections, and cancer. Finally, biopsies may be obtained for histologic assessment. A sigmoidoscopy will come with insufflation so should therefore not be applied in patients with obstruction or toxic megacolon given the increased risk of intestinal perforation.

### Q.3: Which is the role of interventional radiology in the management of intra-abdominal abscesses related to Crohn’s disease in the emergency setting?

#### Statement 3.1

Percutaneous drainage associated with antimicrobial treatment should be considered as a first-line treatment in the management of abscesses related to Crohn’s disease, in stable patients (QoE C).

#### Statement 3.2

Small abscesses (<3 cm) could be treated with intravenous antibiotics with a risk of recurrence, especially if associated with enteric fistula (QoE B)

#### Statement 3.3

Percutaneous drainage of abscesses > 3 cm could avoid immediate surgery and should be used as a bridging procedure before elective surgery to reduce the need for stoma creation and limit intestinal resection in malnourished and high-risk patients (QoE C).

#### Statement 3.4

Surgery should be considered in the case of failure of percutaneous drainage and in patients with signs of septic shock (QoE C).

#### Statement 3.5

Surgery should be considered for patients with enteric fistulae and if clinical evidence of sepsis persists despite the initial treatment plan (QoE C).

#### Recommendations

We recommend performing radiological percutaneous drainage of intra-abdominal abscesses >3cm related to Crohn’s disease associated with early empiric administration of antibiotics, to adapt these as soon as possible to microbiological culture results. Antimicrobial therapy should be re-evaluated according to patient’s clinical and biochemical features (strong recommendation based on a low-level evidence 1C).

We recommend  administering an early empiric antimicrobial therapy in stable patients presenting with abscess <3cm, with close clinical and biochemical monitoring (strong recommendation based on a low-level evidence 1C).

#### Summary of evidence and discussion

Abscess, fistula, bleeding, and stenosis are common complications of CD. Various interventional radiological techniques can be considered as a first-line option for non-operative treatment, with good outcomes. In case of intestinal obstruction for stenosis, bowel dilatation can be performed both with radiological and with endoscopic guidance, in stable patients. Embolization of GI-hemorrhage is technically feasible, but it should be limited to strictly selected cases [35].

Intra-abdominal abscesses in patients with CD typically result from a perforation or penetrating ulcers, and they are an expression of luminal disease activity associated with transmural translocation of bacteria from the diseased bowel to contiguous tissue. Abscesses may be intraperitoneal, retroperitoneal, or intra-mesenteric, most frequently located in the right lower quadrant adjacent to the terminal ileum. In the literature, occurrence rates for intra-abdominal abscesses vary from 10 to 30% [36–39]. Active IBD warrants medical treatment, but the presence of an abscess contraindicates immunosuppressive medication.

There are several treatment options for intra-abdominal abscesses in CD. Previously, the majority of abscesses were treated with operative drainage, but with the improvement of interventional radiological techniques, the use of percutaneous drainage (PD) is increasing, associated with administration of antibiotics.

Success rates for PD in the literature vary from 74 to 100% [36–39]. There are no RCTs comparing percutaneous and surgical drainage, but two meta-analyses tried to clarify the role of percutaneous drainage, compared to surgical management.

He et al. in 2015 [39] aimed to compare clinical outcomes between PD alone and preoperative percutaneous drainage and initial surgery for patients with CD-related spontaneous intra-abdominal abscess, performed a meta-analysis of 9 (non-randomized and retrospective) studies including 513 patients and found a reduction in stoma creation rate and complication rate for patients undergoing pre-operative PD. The reason for these improved outcomes could be related to an improvement in patients’ general and nutritional condition prior to definitive surgical intervention and to the control of the infectious source. Moreover, they reported that the risk for recurrent abscess was higher in patients who underwent PD alone than those who underwent initial surgery, highlighting that delayed abdominal surgery is almost inevitable in the majority of the patients presenting with intra-abdominal abscess.

Another meta-analysis, including six studies with a total of 333 patients was performed by Clancy et al. in 2016 [40]. They compared the use of PD alone and surgery in the management of patients presenting with a CD-related intra-abdominal abscess and reported an increased incidence of abscess recurrence for patients undergoing only PD, but interestingly, they also found that PD can successfully avoid surgery in 29.3% of patients. In addition, they found no significant difference in the overall complication rate, permanent stoma requirement, or length of stay between patients undergoing percutaneous drainage or immediate surgery.

PD (guided by US or CT) appears to be a relatively safe procedure as part of a bridge to surgery technique, but not all abscesses are “drainable” or “accessible.” Effectiveness depends on abscess characteristics, such as location, number, size, presence of fistulae, or close proximity to vital structures.

Independent risk factors for PD failure are bowel wall thickness, disease length, bowel dilation, and abscess size of greater than 6 cm. Furthermore, multiple percutaneous drainage procedures have been required in 8–20% of patients [41] and it could increase the risk of complications such as damage to vital structures in close proximity to the abscess or severe hemorrhage.

Complications of PD, for both spontaneous and post-surgical abscesses (not specific to CD), occur in approximately 10% of procedures. Major complications such as sepsis, small bowel fistulae, colon perforation, and death (due to sepsis or hemorrhage) have been described in 5–11% of cases. Minor complications (such as bacteremia or infection at the site of the catheter insertion) occur in approximately 3% [41].

Feagins et al. [42] suggested that non-drainable abscesses smaller than 3 cm and without evidence of fistula and no steroid therapy are likely to respond to antibiotic therapy alone although with high recurrence rates. In this setting, antibiotics should cover Gram-negative bacteria and anaerobes and it is important to closely observe the clinical condition of the patient in case of any deterioration.

Antibiotic therapy, including a combination of fluoroquinolones or third-generation cephalosporin and metronidazole in patients with CD, should be adapted to the sensitivity of the bacteria (and sometimes fungi) to antibiotics, if PD is performed [43–45]..

The appropriate duration of antibiotic therapy is unclear. Clinical improvement should be seen within 3–5 days after starting antibiotics and percutaneous drainage, with a decrease in drainage production. If a patient’s condition does not improve, re-evaluation and repeat imaging are indicated to determine whether the abscess has been adequately drained. If not, repositioning of the drain or surgical intervention is required [43–45].

If sepsis is controlled after adequate PD, CD medication should be started to prevent recurrence. Patients with a concomitant stenosis, an entero-cutaneous fistula or refractory active disease are likely to require surgery, but preoperative PD, if it is feasible, associated with delayed surgery, can decrease the extent of intestinal resection, postoperative septic complications, and potentially reduce stoma rates.

### Q.4: In patients presenting with complications related to IBD, what is the appropriate medical treatment and nutritional support?

#### a) The role of medical treatment and management of specific IBD drugs

##### Statement 4.1

The optimal management of IBD patients presenting with acute abdominal pain is multidisciplinary, involving a gastroenterologist and an acute care surgeon (QoE C).

##### Statement 4.2

All IBD patients presenting with an acute abdomen should receive adequate volume of intravenous fluids, low molecular weight heparin for thromboprophylaxis and electrolyte abnormalities and anaemia should be corrected (QoE C).

##### Statement 4.3

Antibiotics should not be routinely administered, but only if superinfection is considered and in the presence of an intra-abdominal abscess (QoE B).

##### Statement 4.4

In case of superinfection or abscesses, prompt antimicrobial therapy against Gr/-/aerobic and facultative bacilli and Gr/+/*streptococci* and obligate anaerobic bacilli is needed according to the epidemiology and resistance of the setting. Antimicrobial therapy duration depends on the patient's clinical feature and laboratory test results such as serum CRP level (QoE A).

##### Statement 4.5

The initial medical treatment for severe active UC is intravenous corticosteroids, in case of hemodynamic stability of the patient (QoE A).

##### Statement 4.6

The response to intravenous steroids should be best assessed by the third day (QoE C).

##### Statement 4.7

In non-responder hemodynamically stable patients, medical rescue therapy including infliximab in combination with a thiopurine or ciclosporin should be considered in a multidisciplinary approach (QoE B).

##### Statement 4.8

Infliximab should be considered if anti-inflammatory therapy for penetrating ileocecal Crohn’s disease is required, following adequate resolution of intra-abdominal abscesses in a multidisciplinary approach (QoE C).

##### Statement 4.9

Preoperative treatments with immunomodulators associated with anti-TNF-α agents and steroids are risk factors for intra-abdominal sepsis in patients requiring emergency resectional surgery (QoE B).

##### Statement 4.10

In complex perianal fistulizing disease, infliximab or adalimumab can be used as a first-line therapy in combination with azathioprine following adequate surgical drainage if indicated. A combination of ciprofloxacin and anti-TNF improves short-term outcomes (QoE A).

#### b) The role of nutritional support

##### Statement 4.11

Preoperative nutritional support is mandatory in severely undernourished patients (QoE A).

##### Statement 4.12

Total parenteral nutrition should be reserved for nutritionally deficient IBD patients unable to tolerate enteral nutrition and when the enteral route is contraindicated, in critically ill patients presenting with signs of shock, intestinal ischemia, high output fistula, and/or severe intestinal hemorrhage (QoE B).

##### Statement 4.13

Total parenteral nutrition is the mode of choice when emergency surgery is needed for complicated IBD (QoE A).

##### Recommendations

We recommend evaluating medical treatment in IBD patients presenting with acute abdominal pain and disease activity in a multidisciplinary approach (strong recommendation based on low-level evidence 1C).

We recommend not routinely administrating antibiotics in IBD patients but only in the presence of superinfection, intra-abdominal abscesses, and sepsis (strong recommendation based on high-level evidence 1A)

We recommend administering antibiotics according to the epidemiology and resistance of the setting in a duration that depends on the patient’s clinical and biochemical findings. Antifungals should be reserved for high-risk patients such as those with bowel perforation and recent steroid treatment (strong recommendation based on high-level evidence 1A).

We recommend administering as soon as possible venous thromboembolism prophylaxis with LMWH for the high risk of thrombotic events related to complicated  IBD and the emergency setting (strong recommendation based on high-level evidence 1A).

We recommend weaning off steroids (wean preoperatively, ideally 4 weeks) and stopping immunomodulators associated with anti-TNF-α agents before surgery, as soon as possible to decrease the risk of postoperative complications, in accordance with a gastroenterologist (strong recommendation based on moderate level evidence 1B).

We recommend administering nutritional support (parenteral or enteral, according to GI function and in conjunction with a dietician/nutrition team) in IBD patients as soon as possible (strong recommendation based on moderate level evidence 1B).

##### Summary of evidence and discussion

The management of IBD in an emergency setting is very challenging. Early surgical management is correlated with extended intestinal resection, high stoma rate, and high risk of postoperative complications. Preoperative optimization of the patient, PD, and delayed surgery are associated with decreased risk of complications and length of postoperative stay. Medical treatment should be discussed in a multidisciplinary team. In the management of patients with CD presenting with an intra-abdominal abscess, few studies have addressed medical treatment alone (without percutaneous or surgical drainage) as the primary approach, and all available reports are retrospective or observational studies. According to available data, only abscesses smaller than 3 cm could be treated with antibiotics alone without PD, but it is not clear how to select patients for this therapeutic approach and for how many days to administer antibiotics, with high (37 to 50%) recurrence rates [45].

Following abscess drainage, the preferred CD medical treatment option is anti-TNF therapy in addition to ongoing antibiotic treatment [46]. There are no randomized studies in the literature to clarify whether percutaneous or surgical drainage should always be followed by a delayed resection, although most case series favor a delayed elective resection.

In case of an ileal stenosis, a delayed resection should be considered and it is preferred to de-escalate corticosteroids prior to surgery in order to reduce the risk of post-operative complications [47].

The available literature on the use of biologic therapy before urgent surgery with anti-TNF-α agents, anti-integrin therapy, and anti-interleukin therapy is controversial. Biologic agents can induce and maintain clinical remission, heal the mucosa, and change the natural course of the disease, if used in a timely fashion, avoiding disease progression towards stenosis and fistula formation [48]. The PUCCINI trial [49] is based on a high level of evidence due to the strict protocol followed in collecting data prospectively, and its results demonstrated no effect of anti-TNFs on postoperative complications.

Other factors that can affect postoperative complications in intestinal resections in patients with CD are previous use of steroids, impaired nutritional status, and an unfavorable abdominal environment. Usually, most patients with surgical indication in CD are already using biological agents, and more than one of these factors can also be present. Therefore, in malnourished patients, with previous steroids and/or anemia, the surgical approach can be affected.

In conclusion, direct cause–effect relationship of biologics alone leading to increased rates of complications was not demonstrated and different studies results are controversial [50].

If an urgent surgical exploration is needed, it is extremely important to check concomitant use of steroids, phenotype of the disease, and current nutritional status, in order to establish the surgical plan.

The influence of nutritional status on postoperative morbidity and mortality has been well documented in both retrospective and prospective studies. Poor pre-operative nutritional status has been linked consistently to an increase in post-operative complications and poorer surgical outcome.

Malnutrition is an independent risk factor for adverse postoperative outcomes and affects up to 70% of the IBD population. Malnutrition can occur in UC but is a more common problem in CD since CD can affect any part of the GI tract and UC is restricted to the large bowel, which has few direct malabsorptive effects. Nutritional complications occur in 20 to 85% of patients with CD. This specific condition becomes more serious during the active phase of CD, which is associated with decreased food intake, intestinal absorption dysfunction, drug side effects, and active inflammation [51].

Defining malnutrition is difficult, especially in patients with IBD, and a gold-standard test of malnutrition has not been identified.

The European Society for clinical nutrition and metabolism (ESPEN) reported that serum albumin of <3 g/dl, BMI < 18.5 kg/m^2^, and weight loss > 10–15% within 6 months are the best indicators of severe malnutrition in CD [52].

Parenteral nutrition should be reserved for nutritionally deficient IBD patients unable to tolerate enteral nutrition and when the enteral route is contraindicated in patients presenting with severe shock, intestinal ischemia, high output fistula, and/or severe intestinal hemorrhage [53, 54].

Preoperative nutrition supplementation reduces postoperative complications in patients with CD, in particular, enteral nutrition. In a recent meta-analysis (3 prospective and 2 retrospective studies including 1111 CD patients), it was reported that the rate of postoperative complications in the group receiving preoperative nutritional (enteral or total parenteral nutrition) support was 20.0% compared with 61.3% in the group who had standard care without nutritional support [OR=0.26, 95% confidence interval (CI): 0.07–0.99, *P*<0.001]. Postoperative complications occurred in 15.0% of patients in the group who received preoperative total parenteral nutrition compared with 24.4% in the group who did not (OR=0.65, 95% CI: 0.23–1.88, *P*=0.43). Postoperative complications occurred in 21.9% in the group who received preoperative enteral nutrition compared with 73.2% in the group that did not received preoperative enteral nutrition (OR=0.09, 95% CI: 0.06–0.13, *P*<0.001) [55].

Exclusively enteral nutrition is feasible in CD patients presenting with non-radiologically drainable abdominal abscesses. It is associated with a reduction in surgical rate, optimized preoperative condition, and improved postoperative outcomes in this specific group of patients [56].

Concerning the role of a medical treatment to avoid or postpone surgery, most patients with UC presenting with an ongoing flare in an emergency setting could be treated with intravenous corticosteroids.

In this group of patients, when the checked hemodynamic status is stable, the first step in the diagnostic process includes confirming disease activity with a flexible sigmoidoscopy and ruling out intestinal pathogens including Clostridium difficile and CMV. At this point, it is possible to decide on rescue therapy.

Patients with significant systemic toxicity, as evidenced by severe weight loss, fever, tachycardia, high inflammatory markers, and persisting abdominal pain, should be evaluated for colectomy. If a medical rescue therapy is considered appropriate, IV corticosteroids are administrated. A systematic review of 32 trials of steroid therapy for acute severe colitis, involving 1991 patients from 1974–2006, reported an overall response to steroids of 67% [95% CI 65–69%] [57]. A colectomy was carried out in 29%.

In case of insufficient response to IV corticosteroids, an early (day 3) assessment should take place and subsequent therapy with either infliximab (IFX) (5 mg/kg) or ciclosporin (2 mg/kg/day) should be considered, along with the possibility of surgery. Both medical therapies are potent for inducing remission in this subgroup of patients with comparable outcomes. The open-label CYSIF trial randomized 111 thiopurine-naive patients with severe colitis despite 5 days of IV steroids, to IV ciclosporin 2 mg/kg/day for 8 days followed by 4 mg/kg/day oral therapy, or infliximab 5 mg/kg at weeks 0, 2, and 6 [55]. All responders at day 7 received oral azathioprine and tapered steroids from day 8. Approximately 85% patients in both groups responded to treatment by day 7. Treatment failure at day 98 (the primary endpoint) was reported in 60% patients in the ciclosporin arm compared with 54% patients in the IFX arm. The colectomy rate by day 98 in the ciclosporin vs the infliximab group was 18% vs 21% [*P* = 0.66] [58].

If a surgical procedure is needed, then subsequent steroid withdrawal is mandatory [59]. Antibiotics should not be routinely administered. Controlled trials of oral or IV metronidazole, tobramycin, ciprofloxacin, or vancomycin in acute UC have shown no consistent benefit in addition to conventional therapy [60–62]. In the treatment of complex peri-anal fistulae due to CD an initial abscess drainage and seton placement, according to the symptoms and complexity of the fistula and anti-TNF treatment including infliximab or adalimumab can reduce fistula drainage and induce fistula closure [63, 64].

Antibiotics such as a combination of ciprofloxacin and metronidazole can be added to enhance this effect, but this will only aid in improving short-term clinical outcomes [64]. To enhance the effect of anti-TNF in complex fistulizing disease, combination of anti-TNF treatment with thiopurines should be considered.

Zangenberg et al. [59] carried out a systematic review to identify clear recommendations for the preoperative medical management of patients with IBD, in particular those with CD.

The analysis of the literature showed that before elective surgery:

- Steroid withdrawal (wean preoperatively, ideally 4 weeks unless an emergency) is recommended while steroid stress dose is not recommended;
- Thiopurines’ administration appears to be safe, but it may be prudent to plan the procedure remotely from the last dose of an anti-TNF agent;
- Nutritional risk screening is recommended to unveil and correct any malnutrition;
- Venous thromboprophylaxis prior to surgery is well supported by evidence while extended 4-week prophylaxis needs further research but is likely to be beneficial;
- Percutaneous us or CT-guided drainage for intra-abdominal abscesses is recommended with a considerable risk of recurrence;
- Smoking cessation can be beneficial for wound healing, as well as reducing the risk of disease recurrence in CD.

### Q.5: What are the indications for emergency surgery in patients presenting with complications related to IBD?

Urgent surgical treatment is to be considered in the following clinical setting:

#### 1) Acute severe ulcerative colitis

##### Statement 5.1.1

If a patient’s condition does not improve or deteriorates within 48 to 72 h from initiation of medical therapy, a second-line therapy or surgery should be considered and discussed by the emergency surgeon and the gastroenterologist in acute severe ulcerative colitis (QoE C).

##### Statement 5.1.2

In the event of surgical complications such as free perforation, life-threatening hemorrhage (unstable patients), or generalized peritonitis, immediate surgery is recommended in acute severe ulcerative colitis (QoE B).

##### Statement 5.1.3

In case of no improvement with a second-line therapy, in discussion with a gastroenterologist, surgery is recommended in acute severe ulcerative colitis (QoE C).

##### Statement 5.1.4

Subtotal colectomy with ileostomy is a safe and effective treatment for patients requiring emergency surgery for acute severe ulcerative colitis presenting with massive colorectal hemorrage (QoE B).

##### Recommendations

We suggest evaluating all hemodynamically stable patients  presenting with acute severe ulcerative colitis in a multidisciplinary approach with the gastroenterologist to decide on options for initial medical treatment (weak recommendation based on low-level evidence 2C).

We recommend performing emergency surgical exploration in hemodynamically unstable patients, according to damage control principles and in patients with colonic perforation. A Subtotal colectomy with ileostomy in acute severe ulcerative colitis is the surgical treatment of choice in patients presenting massive colorectal haemorrhage or non-responders to medical treatment (strong recommendation based on high-level evidence 1A).

##### Summary of evidence and discussion

According to the diagnostic criteria of Truelove and Witts for UC severity classification, acute severe colitis affects 5 to 15% of patients with UC and is characterized as colitis with bloody stool frequency ≥ 6/day and a tachycardia (>90bpm), or temperature >37.8°C, or anemia (hemoglobin 10.5g/dL), or an elevated ESR (>30mm/h) (only one additional criterion in addition to the bloody stool frequency ≥6/day is needed to define a severe attack). Endoscopic criteria for severe colitis include a hemorrhagic mucosa with deep ulceration, mucosal detachment on the edge of these ulcerations and well-like ulceration, all of which can be assessed at flexible sigmoidoscopy [65].

There are some controversies regarding the indications for surgery in case of severe acute colitis, and the determination of the correct time for surgery is still a matter of debate. It is well known that the majority with severe acute colitis will respond to medical therapy: in 2007, Turner et al. [57] conducted a systematic review including 32 clinical trials analyzing the response to steroid therapy in severe UC and found that the response rate to steroids is 66% and 34% of patients required colectomy within a short period of time. This study has some limitations, such as the time period of the included studies (1974–2006) and the wide heterogeneity among included cohorts of patients, but interestingly found a small reduction in colectomy rates in the studies that reassessed the need for colectomy after 2 weeks of medical therapy versus those who did it within 2 weeks. Furthermore, the introduction of biologic therapy opened new perspectives for salvage therapy in steroid-refractory patients and some studies demonstrated that up to 80% of patients with acute severe colitis resistant to steroid therapy could respond to biologic therapy, thus avoiding an emergent colectomy [66, 67]. Post-operative morbidity is higher after emergency surgery when compared with elective surgery, for both UC and CD [68], suggesting that the increased use of rescue therapy may contribute to reducing emergency surgical interventions and improve outcome [69]. On the other hand, there is evidence to suggest that both a delay in surgery [70, 71] and prolonged intravenous immunosuppressive therapy are associated with increased morbidity and mortality following subsequent surgery [72, 73]. Based on these results, it seems reasonable to suggest a tailored approach to the patient with acute severe colitis and an attempt at initial conservative management with bowel rest, parenteral nutrition, parenteral steroids, and broad-spectrum antibiotics. The patient must be monitored closely for any signs of progressive deterioration, such as worsening pain or tenderness, progressive leukocytosis, fever, tachycardia, or hypotension. For steroid refractory disease, surgical options or therapeutic alternatives for rescue therapy should be considered early (on or around day 3 of corticosteroid therapy) by a multidisciplinary team that includes both a surgeon and gastroenterologist.

#### 2) Toxic megacolon

##### Statement 5.2.1

In patients presenting with toxic megacolon complicated by perforation, massive bleeding (unstable patients), clinical deterioration, and signs of shock, surgery is mandatory (QoE A).

##### Statement 5.2.2

In patients presenting with toxic megacolon, showing no clinical improvement and biological signs of deterioration after 24–48 h of medical treatment, surgery is mandatory (QoE B).

##### Recommendation

We recommend not delaying surgery in critically ill patients presenting with toxic megacolon (strong recommendation based on moderate -level evidence 1C).

##### Summary of evidence and discussion

Toxic megacolon is a rare but severe and potentially fatal complication of colonic inflammation.

Its main characteristics are as follows [74]:

- Radiographic evidence of total or segmental colonic distention of > 6 cm
- Presence of systemic toxicity
- Inflammatory (or infectious) etiology.

In order to avoid colectomy, medical treatment should be carried out aggressively and in a timely fashion (steroids, fluids, transfusions, etc.) and the correct timing for surgery is still controversial. In fact, while some evidence suggests that the initial medical treatment obviates the need for surgery in about 50% of patients [75], there is a significant body of literature that highlights how a delay in surgical intervention carries the risk of colonic perforation and abdominal compartment syndrome, thus increasing the mortality rate [76, 77]. For these reasons, management of toxic megacolon requires coordination between medical and surgical services with aggressive attempts at medical therapy and early surgical intervention in the absence of improvement, development of complications, or deterioration. Frequent re-evaluations must be performed until the patient’s condition has clearly improved or until there is evidence of deterioration, in which case urgent surgery is indicated. Persistent fever after 48–72 h of steroid therapy should raise the possibility of local perforation or abscess. Free perforation, massive hemorrhage, increasing transfusion requirements, increasing signs of toxicity, and progression of colonic dilatation are indications for an urgent operation.

Unlike colonic obstruction, in which cecal dilation with perforation is a concern, the transverse colon is the area of greatest concern in toxic megacolon. Perforation in patients with toxic megacolon is associated with a high mortality rate (27–57%), regardless of whether the perforation is contained or free [78–80].

#### 3) Uncontrolled gastrointestinal bleeding

##### Statement 5.3.1

Pre-operative localization of the bleeding site, with the aim of excluding an upper gastrointestinal or an anorectal bleeding will allow better planning of the surgical strategy (QoE C).

##### Statement 5.3.2

An upper and lower GI endoscopy should be the initial diagnostic procedure for nearly all stable patients presenting with acute gastro-intestinal bleeding (QoE C).

##### Statement 5.3.3

Computed tomography angiography should be performed in patients with ongoing bleeding who are hemodynamically stable after resuscitation (QoE C).

##### Statement 5.3.4

Surgical treatment is recommended in patients with life-threatening bleeding and persistent hemodynamic instability and in patients with acute severe ulcerative colitis non-responders to medical treatment presenting with massive colorectal hemorrhage (QoE B).

##### Statement 5.3.5

Significant recurrent gastrointestinal bleeding could be an indication for urgent surgery (QoE C).

##### Recommendations

We recommend performing immediate surgery in unstable patients presenting with hemorrhagic shock, and non responders to resuscitation. An intraoperative ileoscopy, if available, could be useful in localizing the bleeding source in patients with Crohn’s disease. In patients presenting with acute severe ulcerative colitis and refractory hemorrhage, non-responders to medical treatment, the surgical treatment of choice is  a  subtotal colectomy with ileostomy, if skills are present (strong recommendation based on low-level evidence 1C).

We suggest evaluating hemodynamically stable IBD patients presenting a gastrointestinal bleeding at first with a sigmoidoscopy and an esophagogastroduodenoscopy (weak recommendation based on low-level evidence 2C).

##### Summary of evidence and discussion

Gastrointestinal bleeding is a common complication in patients with UC or CD and is caused by inflammation/ulceration of the bowel. Usually, the bleeding resolves with medical treatment and rarely it requires an immediate surgical procedure.

Medical treatment includes patients with hemodynamic instability and/or suspected ongoing bleeding receiving intravenous fluid/blood product resuscitation with the goal of normalization of blood pressure and heart rate prior to endoscopic evaluation/intervention. Packed red blood cells should be transfused to maintain the hemoglobin above 7g/dL. A threshold of 9g/dL should be considered in patients with massive bleeding, significant comorbidities (especially cardiovascular ischemia) or possible delay in receiving therapeutic interventions [81].

Massive, life-threatening lower gastrointestinal bleeding is uncommon in patients with IBD and occurs in less than 6% of cases [82]. Because of the low incidence of severe hemorrhage in IBD, a limited number of studies are available to guide management.

In IBD patients, bleeding has different features depending on the underlying disease. In UC, the bleeding typically occurs in patients with pancolitis from diffuse areas of mucosal ulceration. In CD, however, the bleeding most often is a result of focal erosion into an intestinal vessel, and this could include the small bowel.

In UC, the endoscopic assessment and treatment of the bleeding source that could be massive because of the diffuse nature of colonic inflammation is rarely possible. In CD, which is often a segmental disease, it is useful to try to localize the source of bleeding preoperatively to avoid surgery or an extensive intestinal resection, even more so severe bleeding in a patient with CD could be due to an associated condition, such as gastritis or peptic ulcer disease, or multiple segments of GI tract could be involved in the bleeding and this could be a diagnostic and therapeutic challenge for the endoscopist, with a high risk of re-bleeding.

The hemodynamic status of the patient presenting with hematemesis or massive melena or bright red rectal bleeding has to be assessed: the patient has to be resuscitated and stabilized, and a nasogastric tube is inserted to protect the airway and decompress the stomach. In a stable patient, a gastroscopy could be required to rule out an upper gastrointestinal bleeding. If a lower gastrointestinal source is suspected then one should consider sigmoidoscopy or colonoscopy.

The clinical significance of performing contrast-enhanced CT before colonoscopy has been examined in recent years. In a retrospective study of acute LGIB, Nagata et al. reported that the detection rate for vascular lesions was higher for colonoscopy following CT than for colonoscopy alone (35.7% vs 20.6%, *P* = 0.01), leading to more endoscopic examinations (34.9% vs 13.4%, *P* < 0.01) [83].

In patients with IBD who are bleeding, the role of angiography and of angioembolization is not yet clear: the few studies available in the literature are case reports and suggest that angiography/angioembolization could be feasible in stable patients, but further studies are needed to better define outcomes in emergency setting.

The major advantage of angiography and embolization is that it can control severe bleeding without bowel preparation. A systematic review reported that super-selective angiographic embolization achieves immediate hemostasis in 40–100% of diverticular bleeding with occasional rebleeding (15%) [84]. The disadvantages of angiography and embolization include the requirement for active bleeding and the risk of bowel ischemia and the administration of IV contrast. The rate of bowel ischemia following embolization was 1–4% in recent studies [85, 86]. Angiography localizes the bleeding source in 24–70% of cases [87]. Angiography requires blood loss rates > 0.5 mL/min to localize a bleeding site [88].

Moreover, CT angiography may be useful as a noninvasive diagnostic tool prior to angiography, because it is more sensitive and identifies bleeding at rates of 0.3 mL/min [89]. Other methods of localization include the use of a nuclear medicine labeled red cell scans if bleeding is not detected by angiography [65].

Once the source is known, the decision to perform a surgical urgent procedure depends on bleeding source itself, the hemodynamic condition of the patient and on the availability/feasibility of less invasive therapeutic options (i.e., endoscopy or angiography) [90].

If the patient is unstable, even after significant resuscitation, a surgical exploration is mandatory. In clinical practice, surgery is indicated for patients who demonstrate continued hemorrhage despite resuscitation or have other indications for resection of diseased bowel [91].

Significant bleeding is a rare event in Crohn’s disease, and all common causes of upper and lower gastrointestinal bleeding should be assessed; when a surgical exploration is required, it is recommended to perform an intra-operative ileoscopy to find the source of bleeding if the source has not been identified pre-operatively. All efforts to identify the bleeding source should be made pre-operatively.

In case of acute severe ulcerative colitis, the bleeding could involve all the colon mucosa, and the surgical treatment of choice is a subtotal colectomy with ileostomy, to decrease the risk of recurrent bleeding, but it is important to have performed a flexible sigmoidoscopy to make sure there is no significant bleeding source in the rectum.

#### 4) Free perforation

##### Recommendation

We recommend performing surgical exploration in the presence of radiological signs of pneumoperitoneum and free fluid within the peritoneal cavity in acutely unwell patients presenting with complicated Crohn's disease or acute severe ulcerative colitis (strong recommendation based on low-level evidence 1C).

##### Summary of evidence and discussion

Free peritoneal perforation in inflammatory bowel disease is a rare condition, with few cases reported in the literature. It occurs in 1–3% of Crohn’s disease patients as a first manifestation or in the course of the disease [92], and it is more frequent in severe acute ulcerative colitis. Perforation is considered a serious and potentially life-threatening event and is one of the main indications for emergency surgical intervention. Free perforation is the indication for surgery in up to 16% of the cases of complicated IBD [93, 94]. The site of perforation is usually located in the colon in cases of UC, while patients with CD can present with perforation of either the small or large bowel. Free perforation is an absolute indication for emergency surgery, even though the evidence available in the literature is scarce, but delayed surgery is correlated with high mortality and morbidity in patients with IBD.

#### 5) Intestinal obstruction

##### Statement 5.5.1

Surgery is mandatory for symptomatic strictures that do not respond to medical therapy and are not amenable to endoscopic dilatation (QoE C).

##### Statement 5.5.2

Any colorectal stricture should be assessed with endoscopic biopsies to ensure the absence of malignancy (QoE C).

##### Recommendation

We recommend performing surgery in patients presenting with bowel obstruction because of fibrotic or medically resistant stenosis (strong recommendation based on low-level evidence 1C).

##### Summary of evidence and discussion

Small bowel obstruction is the most common complication requiring elective surgery in CD and affects up to 54% of patients [95]. Strictures complicating CD usually affect the small bowel but could arise anywhere in the GI tract and could be divided into inflammatory or fibrostenotic. It is often difficult to distinguish whether the obstruction is caused primarily by inflammation or fibrostenosis. Patients with inflammatory disease deserve a trial of medications aimed at reducing inflammation. Conversely, patients with symptomatic fibrostenotic disease and obstruction require an interventional approach, either surgical or endoscopic.

Endoscopic balloon dilation has proven successful in the management of primary intestinal strictures or anastomotic strictures in CD. For fibrotic strictures, endoscopic balloon dilation has a technical success rate of 89 to 92%, with 70 to 81% patients experiencing short-term relief of symptoms [96]. Long-term results are less impressive, with 73.5% of patients requiring a repeat dilation and 43% requiring surgical intervention within 2 years [97].

Endoscopic stricturotomy is an evolving, novel therapy for which only few short-term retrospective studies exist to demonstrate its safety and efficacy compared with endoscopic balloon dilation or ileocolic resection [95].

Large bowel strictures, especially in UC, should raise high concern for malignancy [98]. When required to perform an emergent colectomy, oncologic principles should be followed. Patients with small bowel stenosis mainly due to inflammation may improve with medical treatment such as steroids. In patients that do not show improvement with medical treatments, suspect stenosis due to fibrosis, and consider radiological investigation with CT or MR enterography and the possibility of endoscopic dilation on the basis of the length and site of the stenosis, the number of stenotic sites, and the presence of ulcers.

Surgery is warranted for small bowel CD stenosis that causes an intestinal obstruction with potential impending perforation, with long or multiple strictures, when the stricture is not endoscopically accessible and when medical and/or endoscopic treatment fails to adequately improve the patient’s symptoms or when there is concern about concomitant malignancy.

### Q.6: Which surgical approach is recommended for complicated IBD in the emergency setting?

#### 1) Emergency surgery for ulcerative colitis

##### Statement 6.1.1

In the setting of free perforation and generalized peritonitis or toxic megacolon, in hemodynamically unstable patient, an open approach is recommended (QoE C).

##### Statement 6.1.2

Both open and laparoscopic approaches are otherwise appropriate in the emergency setting, according to patient's hemodynamic stability and signs of sepsis in complicated ulcerative colitis (QoE C).

##### Statement 6.1.3

A laparoscopic approach (multi-port or in a single incision), if local expertise allows, may reduce length of stay and morbidity in hemodynamically stable patients presenting with complicated ulcerative colitis (QoE C).

#### 2) Emergency surgery for Crohn’s disease

**Clinical scenarios:**

##### a) Intestinal obstruction

**Statement 6.2.1**

If emergency surgery is indicated, a laparoscopic approach to adhesiolysis and bowel resection is recommended if appropriate expertise exists, with care taken to avoid iatrogenic bowel injury in patients presenting with intestinal obstruction for Crohn's disease (QoE C).

##### b) **Bleeding**

**Statement 6.2.2**

If the patient presenting with gastrointestinal bleeding in Crohn's disease is hemodynamically stable and endoscopic and/or interventional radiology measures have been unsuccessful, then a surgical exploration in a laparoscopic (multi-port or in single incision) approach is recommended (QoE C).

**Statement 6.2.3**

If the patient presenting with gastrointestinal bleeding in Crohn's disease is hemodynamically unstable and endoscopic and/or interventional radiology procedures have been unsuccessful, then a surgical exploration in an open approach is recommended to reduce operating time (QoE C).

##### c) **Free perforation and purulent/fecal peritonitis**

**Statement 6.2.4**

A laparoscopic approach with resection, lavage and stoma is suggested in hemodynamically stable patients presenting with perforation and peritonitis in Crohn's disease,  to avoid complications associated with anastomotic leak (QoE C).

**Statement 6.2.5**

If there is hemodynamic stability and only localized contamination, an anastomosis may be considered but other factors will also need to be considered (QoE C).

**Statement 6.2.6**

If evidence of severe sepsis/septic shock, damage control surgery may be considered, with resection, stapled off bowel ends, and temporary closure (laparostomy) with return to theater in 24–48 h for a second look, washout, and consideration of stoma vs anastomosis (QoE C).

##### d) Crohn’s colitis

**Statement 6.2.7**

Subtotal colectomy and ileostomy are the emergency operations of choice for severe acute and refractory colitis, with open and laparoscopic approaches appropriate in the emergency setting according to patient's hemodynamic stability (QoE C).

**Statement 6.2.8**

A laparoscopic approach, if local expertise allows, may reduce length of hospital stay and risk of infectious complications (QoE C).

**Statement 6.2.9**

There is insufficient evidence to recommend SILS or robotic surgery in the emergency setting (QoE C).

#### 3) Anastomotic considerations in emergency surgery for Crohn’s disease

**Statement 6.3.1**

If a patient in the emergency setting has 2 or more risk factors for anastomotic complications (such as discussed in the text), then a stoma should be formed following resection (QoE C).

**Statement 6.3.2**

If a decision to anastomose has been made, there is no evidence to suggest that one type of anastomosis (stapled vs hand sewn) is superior to the other in terms of complication rates or recurrence, and the decision can be left to surgeon preference (QoE C).

**Recommendations**

We recommend performing a surgical exploration by laparotomy in a hemodynamically unstable patient presenting with complications related to IBD such as perforation and severe peritonitis, massive intestinal bleeding, obstruction, toxic megacolon, severe colitis non responder to medical treatment,  taking into consideration damage control surgery principles with or without an open abdomen (strong recommendation based on low-level evidence 1C).

We recommend performing a laparoscopic approach in hemodynamically stable patients presenting complications related to IBD, when skills are available, in order to decrease morbidity and length of hospital stay (strong recommendation based on low-level evidence 1C).

We recommend performing a subtotal colectomy with ileostomy in patients presenting with acute severe refracrory colitis, non responders to medical treatment, in a laparoscopic or open approach according to patient’s hemodynamic stability (Strong recommendation based on low level evidence 1C). We suggest considering an (stapled or hand sewn) anastomosis in hemodynamically stable patients with Crohn's disease who have good pre-existing nutritional status and who are taking no steroids or other immunosuppression and presenting with no bowel vascular compromise and only localized peritonitis. A defunctioning stoma should also be considered in the emergency setting (weak recommendation based on low-level evidence 2C).

**Summary of evidence and discussion**

Surgical treatment can be particularly challenging when patients with IBD present as an emergency, as they are often much more unwell than when being considered for elective intervention. Moreover, the surgical options may be more limited and there may also be less time for full multidisciplinary involvement in the decision making process, especially in critically ill patients. In addition, many centers will have an emergency surgical on-call rota that is staffed with surgeons whose main specialist elective practice is not in IBD surgery. Therefore, it is necessary to be aware of the principles of emergency operative surgery for patients presenting acutely with the complications or sequelae of UC and CD.

**Emergency surgery for Ulcerative colitis**

The indications for emergency surgery in ulcerative colitis include medically resistant disease, bleeding, toxic megacolon, and perforation [96, 99]. Open and laparoscopic approaches may be considered in the emergency setting, according to availability of trained local expertise.

There is a role for open surgery [100–103]. An open approach is likely to be favored in the setting of a perforation, where a faster operation is required to allow the patient to have their source control surgery in a timely fashion and to be cared for post-operatively in the ICU as quickly as possible. In addition, toxic megacolon, which is now thankfully uncommon, may prove particularly challenging in terms of handling the bowel laparoscopically without perforation, and so open surgery is again recommended.

Otherwise, laparoscopic surgery has been shown to lead to a reduction in length of stay and infectious complications in the emergency setting [104–106]. The existing data relate to multiport laparoscopic surgery, and as such, this would be recommended over a single port approach in the emergency setting, with no data available to support the use of a robotic approach.

**Emergency surgery for Crohn’s disease**

Indications for emergency abdominal surgery in the setting of Crohn’s disease include bowel obstruction, perforation, and bleeding [96, 99]. Management of patients with an intra-abdominal abscess has been dealt with elsewhere in this guideline.

Up to 16% of patients with Crohn’s disease will present with a bowel perforation [107]. Management decisions are often complex and will need to be made around mode of surgical access, decision for anastomosis, and if so, using which anastomotic technique. Bowel resection is generally required in the emergency setting, with the requirement for strictureplasty being more common in the elective setting. The type of resection needed will be according to the disease site, and this may be a small bowel resection, ileocecal resection, or colonic resection, generally subtotal colectomy.

In terms of mode of access, for the patient with bowel obstruction due to non-active stricturing disease or adhesions, a laparoscopic approach is preferred, according to local expertise, as there is evidence for reduced length of stay and fewer complications [108, 109]. Great care is needed on establishing pneumoperitoneum, as the bowel will be distended, and as such, an open port insertion technique should be used. Available data relate to multi-port laparoscopic approach, as opposed to single-port, and there are no data to support a robotic approach in this setting.

An open approach remains preferable in the setting of bleeding with hemodynamic instability where endoscopic and interventional radiology techniques have not been successful, and in a patient with a free perforation. When a patient with a free perforation has severe sepsis or septic shock, and associated significant peritoneal contamination, it may be preferable to perform damage limitation surgery in the form of bowel resection with stapled off bowel ends, peritoneal lavage, laparostomy, and rapid return to ICU for on-going care, with a planned second look laparotomy 24–48 h later with further bowel inspection, peritoneal lavage, abdominal closure, and consideration of stoma formation versus anastomosis.

**Anastomosis decision making in Crohn’s disease in the emergency setting.**

In the emergency setting, the decision whether to perform a primary anastomosis following resection for Crohn’s disease can be challenging, and there are also considerations of whether a particular anastomotic technique may be preferable if so.

It is imperative to consider the clinical state and presentation of the patient, as well as the indication for surgery, when thinking about whether an anastomosis may be appropriate in the emergency setting. Factors to consider when contemplating anastomosis include the following:

- **Patient factors:** e.g., sepsis, degree of peritoneal contamination (local only vs widespread), hemodynamic stability, need for inotropes, nutrition/albumin, abscess, immunosuppression (e.g., steroids, timing of recent anti-TNF treatment), smoking
- **Disease factors:** fistulising/perforating vs stenotic, proximal jejunal vs ileal

If a patient has 2 or more risk factors in the emergency setting, then a resection with stoma should be performed, rather than an anastomosis. A stoma following resection may still be an appropriate option when only one risk factor is present. If an anastomosis is performed, consideration should be given to a defunctioning stoma.

If an anastomosis (usually small bowel or ileocolic) is to be performed, then in general, the options to consider will include the configuration (end-to-end vs side-to-side) and material (stapled vs suture) used. Overall, in the specific setting of IBD surgery, there is no evidence for superiority of any one technique over the other in terms of complication rates or recurrence [110–112]. Therefore, the decision can generally be left to surgeon preference.

If a stapled anastomosis is being performed, consideration should be given to performing this in an isoperistaltic configuration, in order to allow easier neo-terminal ileal intubation at subsequent surveillance colonoscopy.

If there is chronically thickened small bowel in the setting of a long-standing bowel obstruction, consideration should be given to performing a hand-sewn anastomosis as the stapler may not function adequately in this setting where the bowel wall is very thickened and edematous [112].

Data for the newer Kono-S small bowel anastomotic configuration [113], which is a hand sewn technique that leads to a wide lumen with mesenteric exclusion, are confined to the elective setting and as such, would not be recommended for emergency cases at present.

In Crohn’s colitis, the indications for emergency surgery are as for ulcerative colitis, including medically resistant disease, bleeding, toxic megacolon, and perforation and may also include large bowel obstruction if there is a stricture present. Open and laparoscopic approaches are appropriate in the emergency setting, according to local expertise.

### Q.7: How to manage perianal sepsis in the emergency setting?

#### Statement 7.1

An acute abscess should be adequately drained under general anesthetic, with no routine requirement for wound packing (QoE C).

#### Statement 7.2

No active attempt should be made to find an associated anal fistula at the initial abscess presentation (QoE C).

#### Statement 7.3

If an obvious fistula exists (without probing), the fistula should not be laid open and a loose draining seton should be inserted (QoE C).

#### Statement 7.4

There is no role for any additional surgical fistula treatment modality in the emergency treatment of Crohn’s perianal sepsis (QoE C).

#### Statement 7.5

An assessment of the rectum should be made at the time of abscess drainage, to assess for signs of proctitis (QoE C).

#### Recommendation

We recommend performing adequate surgical drainage of perianal abscess in Crohn's disease without searching for an associated fistula (strong recommendation based on low-level evidence 1C).

#### Summary of evidence and discussion

Around 1 in 3 patients with CD will develop perianal manifestations of the disease [114, 115], which will include the risk of perianal abscess and fistula. When presenting with an acute perianal or ischiorectal abscess, the main principle of surgical intervention is to perform adequate drainage, and this will likely require a general anesthetic in most cases.

In the acute setting, it can be difficult to find an underlying fistula with significant induration and sepsis present, and so no active attempt should be made to find an associated fistula and minimal tissue disruption/destruction should occur. Over-vigorous attempts to probe for a fistula at emergency surgery may lead to an iatrogenic track and internal opening, which will add great complexity to the on-going management of the patient’s perianal disease and greatly increase the risk of non-healing.

If there is a deep cavity evident, consideration may be given to short-term use of an appropriate drain (e.g., corrugated drain, Malecot catheter). In addition, if there is concern of undrained sepsis, potentially being fed from a supralevator source, then an urgent inpatient MRI scan will be required.

There is no requirement for wound packing following abscess drainage [116], although its role is currently under investigation in a multicenter randomized controlled trial in the UK [117]. Packing may have a limited role for short-term hemostatic requirements.

If an associated anal fistula is obvious at the time of abscess drainage without any probing, then a loose draining seton should be inserted. The loose draining seton should be low profile and made of a soft material of the surgeons choice, avoiding any bulky knots and avoiding any firm suture material such as nylon. There should be no attempt to lay the fistula open at the same time, in order to minimise tissue disruption and preserve future anal function.

This approach will allow subsequent treatment planning with the patient, then being an active participant in the multidisciplinary management decisions required to minimize future symptoms and maximize the chance of potential fistula healing [118]. When sepsis is present, there is no role for attempts at surgical adjuncts to fistula healing, such as fibrin glue, fistula plug, LIFT, advancement flap, VAAFT, FiLac, and stem cells, in the emergency setting.

In order to plan future treatment with the patient and IBD multidisciplinary team, it is essential to know if there is associated active proctitis in association with the perianal sepsis. For this reason, an assessment of the rectal mucosa should be made at the time of abscess drainage, either in the form of rigid or flexible sigmoidoscopy.

## Conclusions

Complicated inflammatory bowel disease requires a multidisciplinary approach because of the complexity of this group of different diseases in the urgent/emergency setting with the aim of obtaining good functional outcomes and to decrease stoma rates where possible.

These guidelines represent an effort to summarize high-quality evidence and expert opinion to help acute care surgeons and emergency surgeons in managing an acute abdomen or perineum in patients presenting acutely with complications of UC and CD.

## Data Availability

Not applicable.

## Abbreviations

IBD: Inflammatory bowel disease
UC: Ulcerative colitis
CD: Crohn’s disease
ESR: Erythrocyte sedimentation rate
WBC: White blood cell count
FC: Fecal calprotectin
PCR: Polymerase chain reaction
CMV: Cytomegalovirus
US: Ultrasonography
CT: Computed tomography
MRI: Magnetic resonance imaging
MRE: Magnetic resonance enterography
POCUS: Point of care ultrasonography
CTE: Computed enterography
PD: Percutaneous drainage
GI: Gastro-intestinal
LMWH: Low molecular weight heparin
SILS: Single-incision laparoscopic surgery
WSES: World Society of Emergency Surgery
AAST: American Association for the Surgery of Trauma
